# Supplementary material for: Levels of Amyloid Beta (Aβ) Expression in the Caenorhabditis elegans Neurons Influence the Onset and Severity of Neuronally Mediated Phenotypes
Source: Cells. 2024 Sep 23;13(18):1598. doi: 10.3390/cells13181598 (PMC11430350; doi:10.3390/cells13181598)
Supplement: Supplementary file 1 [file cells-13-01598-s001.zip › cells-2588434-supplementary.pdf]

Table S1: List of primers used in the study.

Gene name	Forward Primer (5′-3′)	Reverse Primer (5′-3′)	Amplicon size (bp)
rgef-1 promoter	GAATCTGCAGCGATCAACTGAAATCCG	TAATCCCGGGCGTCGTCGTCGTCGATGC	2670
snb-1 promoter	GGCGTCTAGAAAA-TATTAATTTATGATGTCAA	ATTTCCCGGG-GATGTCGTCAA-GATGGTCTTA	2023
Y45F10D.4	CGGAACCCGCGAAATGTCGGA	CGGTT-GCCAGGGAA-GATGAGGC	191
cdc-42	CTGCTGGACAG-GAAGATTACG	CTCG-GACATTCTCGAATGAAG	111
hu-DA-Aβ1-42	AGAATTCCGACATGACTCAGG	CACCATGAG-TCCAATGATTGC	108

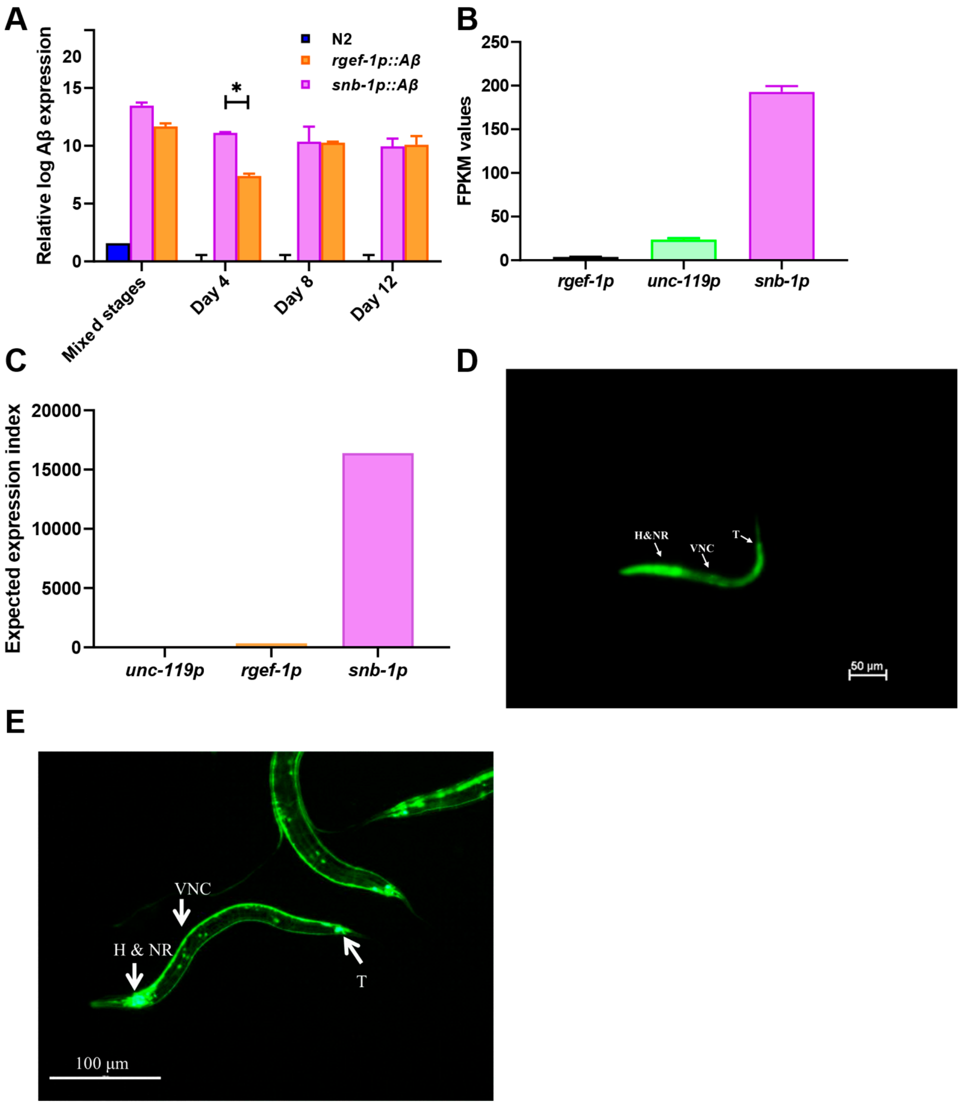

Figure S1: Variation in pan-neuronal promoter expression chosen for this study. A) Comparison of relative log *Aβ* expression at the transcript level in the nematodes, normalised to reference genes *Y45F10D.4* and *cdc-42* driven by *rgef-1* and *snb-1* promoters in young (Day 4), middle-aged (Day 8), Old (Day 12) adults and mixed life stages (N = 3 for mixed life stages and N = 2 for timepoints day 4, day 8, and day 12 consisting of 200-300 worms per replicate) *p < 0.05, ***p < 0.01. An unpaired t-test was used to test for significant differences in relative *Aβ* expression between the two transgenic *C. elegans* strains *rgef-1*p::*Aβ* and *snb-1p::Aβ*. B) Plot of FKPM values of the selected pan-neuronal promoters using data derived from Worm base. C) Bar graph of expected expression index for three pan-neuronal promoters used in the study. Expected expression index was calculated by multiplying the *Aβ* transgene copy number and the FKPM value. D) Transgenic *C. elegans* larval stage 2 (L2) worm showing pan-neuronal GFP expression driven by *snb-1* promoter fragment. GFP expression is evident in the head and tail neuron regions. The GFP expression on the body of the worm indicates the network of nerves running along the length of the nervous system (Scale bar = 50 µm). E) Pan neuronal GFP expressing transgenic *C. elegans* larval stage 4 (L4) worm driven by *rgef-1* promoter. GFP expression is evident in the head and nerve ring (H&NR), Dorsal and Ventral nerve cord (VNC) and Tail neurons (T) (Scale bar = 100 µm). All bar graph data are reported as mean ± standard error of the mean (SEM).

Table S2: Summary of lifespan experiments conducted.

Study	Strain name	Median LSP (days)	Maximum LSP (days)	# worms that died/Total
1	N2	19	32	110/120
	mCherry control	18	30	100/120
	snb-1p:: Aβ1-42	13	19	107/120
	rgef-1p::Aβ1-42	13	22	103/120
	YFP control	16	28	119/120
	unc-119p::Aβ1-42	15	22	112/120
2	N2	19	32	110/120
	mCherry control	18	32	102/120
	snb-1p:: Aβ1-42	13	22	93/120
	rgef-1p::Aβ1-42	15	19	81/120
	YFP control	16	28	104/120
	unc-119p::Aβ1-42	15	22	86/120
3	N2	20	32	110/120
	mCherry control	17	30	107/120
	snb-1p:: Aβ1-42	13	20	90/120
	rgef-1p::Aβ1-42	15	24	97/120

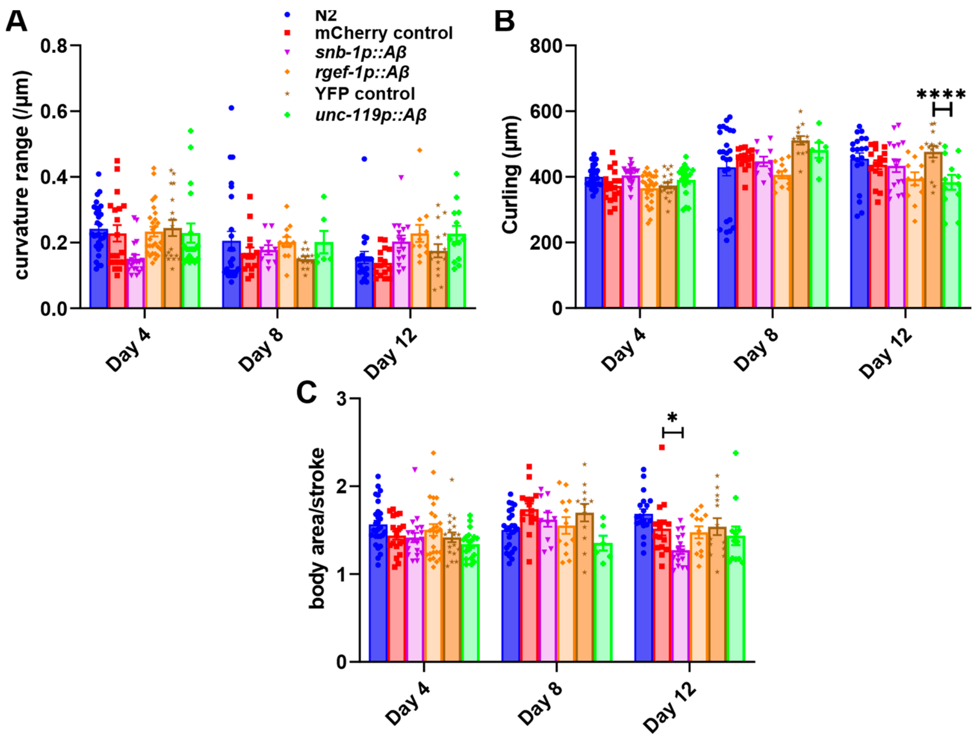

Figure S2: Measurement of motility parameters of the transgenic *C. elegans* strains in liquid media. A) Dynamic amplitude (/µm), B) Curling (µm) C) Brush stroke (body area/s) (n = 2, 5-15 worms/replicate). All data analysed by two-way ANOVA followed by post hoc Tukey multiple comparisons test. The *snb-1p::Aβ* and *rgef-1p::Aβ* have been compared to the transgenic mCherry control strain and the *unc-119p::Aβ* has been compared to the transgenic YFP control strain. *p < 0.05, ****p < 0.0001. All bar graph data are reported as mean ± standard error of the mean (SEM). The genotypes shown in the key in Panel A are the same across multiple panels.

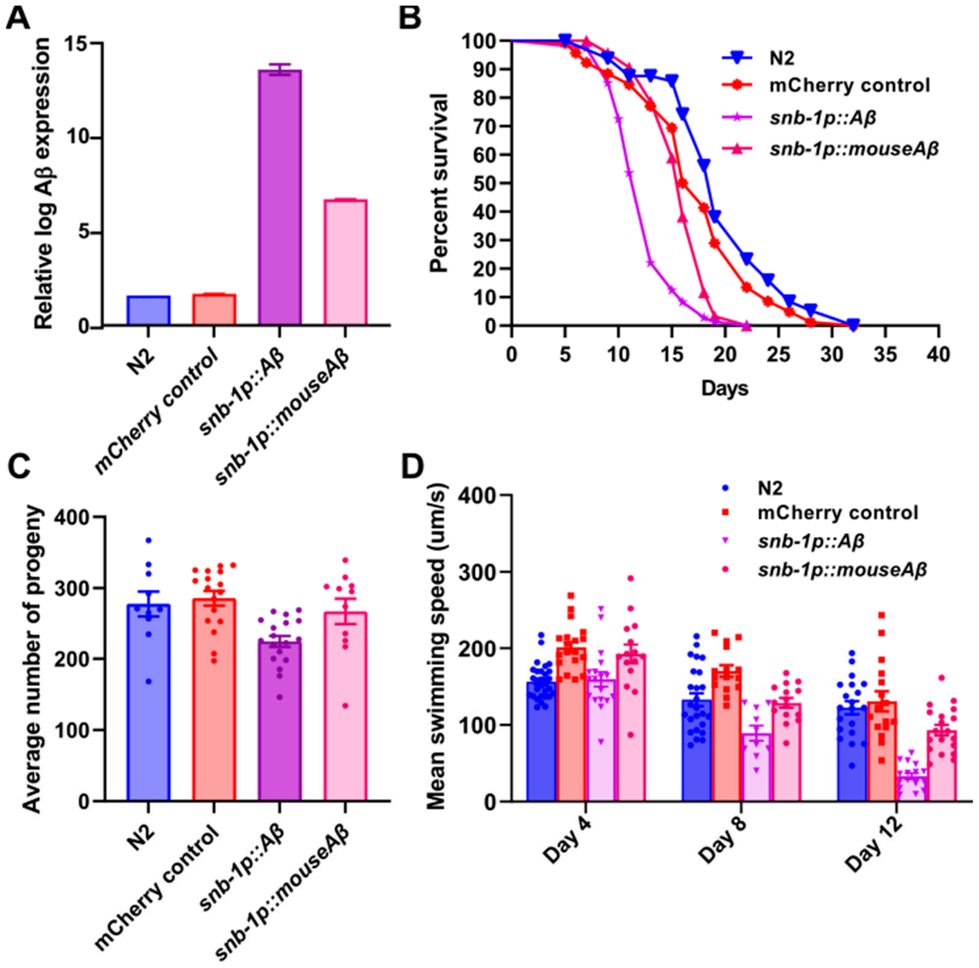

Figure S3: Molecular and behavioural phenotyping of mouse *Aβ*-expressing strain in comparison to the human *Aβ*-expressing strain. A) Relative log *Aβ* expression at the transcript level in the nematodes (mixed life stages), normalised to reference genes *Y45F10D.4* and *cdc-42* (N = 3 replicates, n = 200 – 300 worms/replicate). B) Representative Kaplan-Meier curve of one biological replicate (n = 120). C) Total brood size (n = 3, 5-7 worms/replicate). D) Mean swimming speed (μm/s). All bar graph data are reported as mean ± standard error of the mean (SEM).
